# Enhancing ACL reconstruction: augmented hamstring allograft with high-strength sutures for superior graft stability

**DOI:** 10.1093/jscr/rjaf013

**Published:** 2025-04-21

**Authors:** Danaithep Limskul, Rattanavit Phandthong, Napatpong Thamrongskulsiri, Thun Itthipanichpong, Thanathep Tanpowpong, Somsak Kuptniratsaikul

**Affiliations:** Department of Orthopaedics, Faculty of Medicine, Chulalongkorn University and King Chulalongkorn Memorial Hospital, The Thai Red Cross Society, 1873 Rama IV Road, Pathumwan, Bangkok 10330, Thailand; Division of Academic Affairs and Chulalongkorn University International Medical Program (CU-MEDi), Faculty of Medicine, Chulalongkorn University, 1873 Rama IV Road, Pathumwan, Bangkok 10330, Thailand; Division of Academic Affairs and Chulalongkorn University International Medical Program (CU-MEDi), Faculty of Medicine, Chulalongkorn University, 1873 Rama IV Road, Pathumwan, Bangkok 10330, Thailand; Sports Medicine Research Group, Faculty of Medicine, Chulalongkorn University, 1873 Rama IV Road, Pathumwan, Bangkok 10330, Thailand; Department of Orthopaedics, Faculty of Medicine, Chulalongkorn University and King Chulalongkorn Memorial Hospital, The Thai Red Cross Society, 1873 Rama IV Road, Pathumwan, Bangkok 10330, Thailand; Department of Orthopaedics, Faculty of Medicine, Chulalongkorn University and King Chulalongkorn Memorial Hospital, The Thai Red Cross Society, 1873 Rama IV Road, Pathumwan, Bangkok 10330, Thailand; Department of Orthopaedics, Faculty of Medicine, Chulalongkorn University and King Chulalongkorn Memorial Hospital, The Thai Red Cross Society, 1873 Rama IV Road, Pathumwan, Bangkok 10330, Thailand

**Keywords:** ACL, allograft, knee, augmentation, arthroscopy

## Abstract

The conventional autograft technique for anterior cruciate ligament (ACL) reconstruction poses challenges such as donor site morbidity and postoperative pain. This technique introduces an augmented allograft technique using high-strength sutures to enhance graft stability and healing. The method involves preparing a semitendinosus and gracilis allograft. This technique reduces donor site morbidity, preserves the patient’s natural anatomy, and may allow for faster rehabilitation. Additionally, it decreases the chance of contralateral injuries and graft re-rupture, enhancing ACL strength and knee stability. The augmented allograft with high-strength suture presents a promising alternative for ACL reconstruction, offering increased graft strength and knee stability while avoiding complications associated with autograft harvesting.

## Introduction

The conventional autograft technique remains the recommended guideline for anterior cruciate ligament (ACL) reconstruction. However, alternative methods, including allografts, have emerged in response to concerns regarding donor site morbidity, postoperative pain, potential loss of knee function, and larger incision sites associated with autografts [1, 2]

A central argument concerning allografts is their potential inferiority as some studies indicate that allografts may exhibit higher graft failure rates [3]. This issue can be attributed primarily to two factors: graft strength and re-rupture rates. It is noteworthy that many previous studies on allografts have focused on their application without augmentation [4–8]. To target the graft strength issue, there has been a growing body of literature addressing the use of bio-composite scaffolds and high-strength sutures to enhance allograft performance [9, 10].

Augmented allografts integrate the benefits of allograft tissue with additional biological or synthetic materials to improve graft stability and promote healing. In this context, we propose the use of a high-strength suture technique, utilizing ultra-high-molecular-weight polyethylene (UHMWPE) sutures, as a method for augmenting ACL allografts during reconstruction. This approach aims to leverage the advantages of augmented allografts while addressing the inherent challenges associated with traditional allograft techniques.

## Case report technique

**Figure 1:**
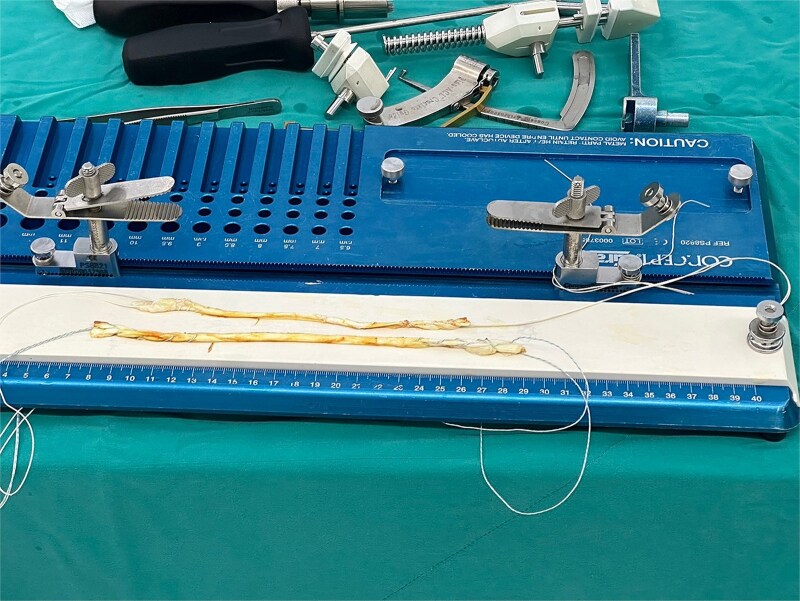
Hamstring allograft with high-strength suture whipstitched at both ends of the graft. The same color of suture is used for each graft for identification of the graft.

In this demonstration, we begin by preparing a semitendinosus and gracilis allograft by leaving the graft at room temperature and soak with 7.5% povidine solution mixed with normal saline solution (NSS) in a proportion of 1:1 for 15 min. After soaking the solution, the graft is then rinsed with NSS and both ends of each tendon were whipstitched using No. 2 Hi-Fi suture (ConMed, Utica, NY). The same color of the suture is used for the other end of the same graft to allow identification of the graft. Each pair of sutures from the allograft is tied to itself for further identification when the sutures are managed at the end of the tibia (Fig. 1).

**Figure 2:**
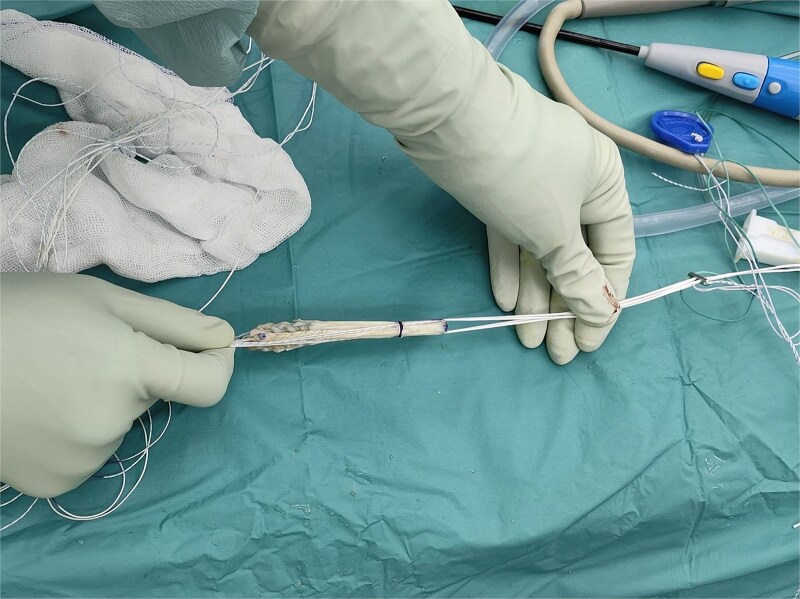
Hamstring allograft with high-strength suture augmentation. The sutures run beside the allograft.

The graft is then applied to a cortical loop fixation system. For this demonstration, we use Infinity adjustable loop cortical fixation (ConMed, Utica, NY) for femoral fixation. After that, the allograft is tensioned at 20 pounds for 20 min to prevent further intraarticular creeping of the graft. Then, two No. 2 Hi-Fi sutures are used to augment the allograft (Fig. 2).

The knee joint is prepared and the femoral tunnel is drilled and reamed in a conventional fashion at the femoral footprint of the ACL. A No. 2 Ethibond is passed through the femoral tunnel by the femoral pin and resides in the femoral graft, with the loop on the outside of the knee, for further graft insertion. After the femoral tunnel preparation is completed, the tibial tunnel is then prepared using a tibial guide. A small incision is done at the anteromedial aspect of the proximal tibia for insertion of the tibial guide for further pin insertion. This incision at the tibia will be used for further suture-to-post fixation as well. After the tibial footprint has been located, the pin is then inserted and reamed accordingly, with the size equivalent to the graft. The loop-end of the Ethibond in the femoral tunnel is used for allograft delivery through the tibial tunnel. The button is shuttled and positioned at the lateral aspect of the femoral cortex. Next, the augmented allograft is pulled into the reamed femoral tunnel. After that, an interference screw is inserted for tibial fixation. For our demonstration, we use Genesys Matryx (ConMed, Utica, NY) for tibial fixation.

**Figure 3:**
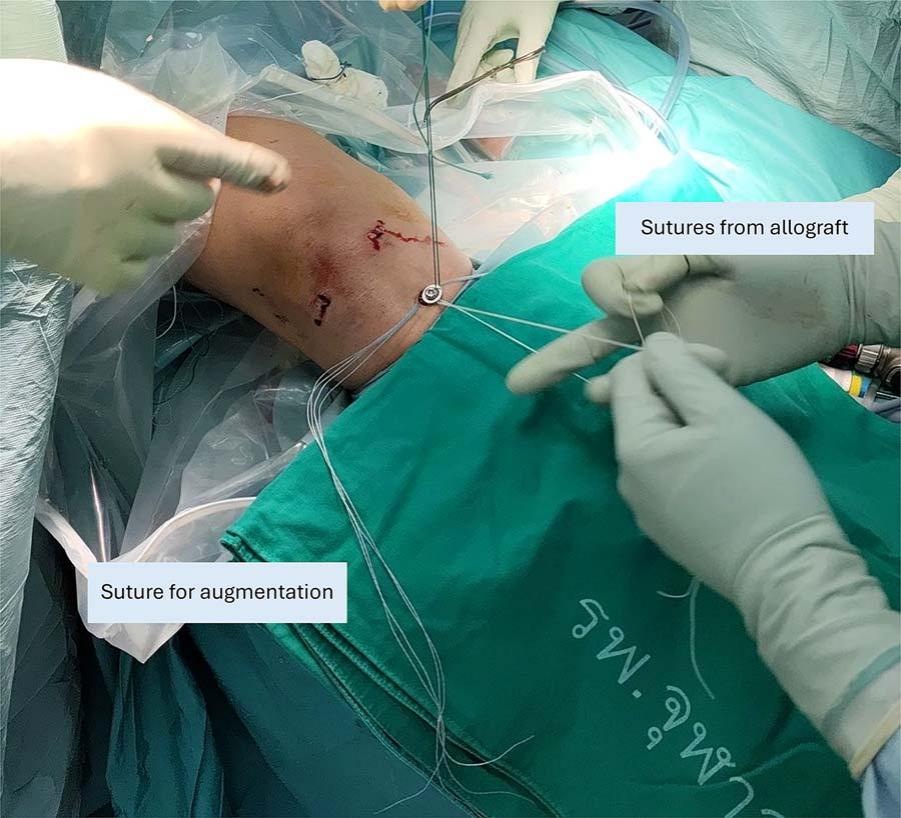
Suture-to-post fixation at the proximal tibia. The sutures from the graft ends are being tied. The sutures for augmentation are tied afterwards.

A 4.5-mm cortical screw with washer is applied 2 cm distally from the tibial tunnel for suture-to-post fixation. The remaining sutures from each allograft, which can be distinguished from the knot, are passed and tied around the screw. Finally, the sutures for augmentation are tied together around the screw (Fig. 3).

### Augmentation material

The Hi-Fi suture was used to augment the ACL hamstring allograft. This suture is a braided, non-absorbable surgical suture constructed from ultra-high molecular weight polyethylene (UHMWPE) fibers. UHMWPE is a biocompatible material known for its exceptional tensile strength, minimal elongation, and resistance to wear and fatigue. These properties make it particularly suitable for orthopedic applications where high durability is essential. The braided design of the Hi-Fi suture enhances knot security and minimizes the risk of slippage, contributing to the overall stability of the augmented allograft. Additionally, the smooth surface and flexibility of UHMWPE fibers reduce tissue trauma during insertion and handling, facilitating precise surgical outcomes [11]. By incorporating this advanced suture material, the technique aims to reinforce graft strength and stability, reducing the risk of re-rupture and promoting more robust biomechanical performance during early rehabilitation.

## Discussion

For our technique, we focused on the augmented allograft with high-strength sutures made from UHMWPE, which utilizes the hamstring allograft. There are several notable advantages with this technique:

### 1) Comparing augmented allograft vs conventional autograft

#### Tissue preservation and host morbidity

The technique eliminates the need for surgeons to harvest tissue from a healthy donor site, thereby completely avoiding donor site morbidity and associated functional loss.

#### Reducing complications

A recent large case series, which studied a total of 2915 ACL-reconstructed patients who underwent either allograft or autograft, indicates that patients who underwent autograft procedures experienced a higher rate of contralateral injuries at 7.2 and 3.9% for allograft group and autograft group, respectively, while those receiving allografts had an increased risk of re-rupture [12]. Notably, the contralateral rupture rate was higher than the revision rate. The use of augmented allografts may effectively address these concerns by potentially reducing the incidence of contralateral knee injuries while also decreasing the likelihood of allograft rupture.

#### Faster rehabilitation

Traditional autograft techniques often require a longer recovery period due to the additional trauma inflicted on the donor site, which can lead to increased pain and extended healing times. In contrast, the augmented allograft technique eliminates the need for harvesting tissue from a healthy donor site, thereby reducing overall surgical trauma and postoperative pain.

The use of high-strength sutures to augment the allograft enhances the initial stability of the graft, which is crucial for early mobilization and rehabilitation. This increased stability allows patients to begin range-of-motion exercises sooner, which are essential components of a successful rehabilitation program. Early mobilization helps in maintaining muscle strength, preventing joint stiffness, and promoting faster recovery of knee function [13].

### 2) Impact of augmentation of ACL allograft to conventional allograft

Multiple studies have demonstrated that augmentation significantly decreases graft rupture rates compared to conventional allografts. The use of UHMWPE sutures provides greater load-sharing capabilities, which mitigates stress on the allograft tissue and enhances overall structural integrity [4, 5].

#### Increase in ACL strength and knee stability

There are studies that use multi-strength suture to enhance ACL repair [10]. For the purpose of smaller knot at the suture-to-post tibial fixation, comparing with the conventional allograft, augmentation with two No. 2 Hi-Fi sutures will increase the load sharing between allograft and the high strength suture. As a result, it will decrease one of the main risk factors of graft ruptures that are caused by graft strength issues.

#### Return-to-sport timelines

Augmented allografts with UHMWPE sutures have shown promising results in reducing the time required for athletes to safely return to sport. Enhanced graft stability minimizes the risk of reinjury during early rehabilitation phases, supporting quicker recovery and functional restoration [14].

#### Complications and safety profile

Complication rates associated with augmented allografts are generally low. UHMWPE sutures exhibit high tensile strength and biocompatibility, which contribute to a favorable safety profile and reduced risk of graft elongation or failure over time. These factors underscore the technique’s efficacy and reliability in ACL reconstruction [15].

## Conclusion

The augmented allograft with high-strength suture is an innovative approach to ACL reconstruction that aims to increase graft strength and provide knee stability. Patients can have the option to not harvest their own healthy tissue to compensate for the ACL loss. Future studies with long-term follow-up are warranted to further validate the benefits and safety of this method in diverse patient populations.
